# Novel Adhesion Technique Using Metallic or Non-Metallic Hydrous Oxide of Metal Complexes Involving Magnetic Compound Fluid Rubber under Electrolytic Polymerization and Magnetic Field for Producing Sensors

**DOI:** 10.3390/s19030689

**Published:** 2019-02-08

**Authors:** Kunio Shimada, Hiroshige Kikura, Hideharu Takahashi, Ryo Ikeda

**Affiliations:** 1 Faculty of Symbiotic Systems Sciences, Fukushima University, 1 Kanayagawa, Fukushima 960-1296, Japan; 2 Institute of Innovative Research, Tokyo Institute of Technology, 2-12-1 Ookayama, Meguro-ku, Tokyo 152-8550, Japan; kikura@lane.iir.titech.ac.jp (H.K.); htakahashi@lane.iir.titech.ac.jp (H.T.); ikeda.r.ah@m.titech.ac.jp (R.I.)

**Keywords:** metallic hydrous oxide, non-metallic hydrous oxide, metal complex, natural rubber, chloroprene rubber, adhesion, electrolytic polymerization, magnetic cluster, magnetic field, magnetic compound fluid (MCF), sensing, artificial skin, robot

## Abstract

As per sequential studies on new types of soft rubber for the artificial skin of robots, smart sensors, etc., we have proposed and investigated hybrid skin (H-Skin) and haptic sensors by using magnetic compound fluid (MCF), compounding natural rubber latex (NR-latex), and applying electric and magnetic fields. Through electrolytic polymerization, the MCF rubber is solidified. The MCF rubber has hybrid sensing functions and photovoltaic effects, and electric charge as battery. In case of the production of soft rubber sensors, however, the problem of adhesion between metal electrodes and rubber is very important. In the present study, we propose a novel adhesive technique for bonding the metal electrodes and MCF rubber by using metallic or non-metallic hydrous oxide, which is a metal complex, via electrolytic polymerization. The anionic radical hydrate reacts with the isoprene molecules of NR-latex or chloroprene rubber latex (CR-latex) such that they are cross-linked and the MCF rubber with the hydrate is solidified, which can be represented via a chemical reaction equation. By means of this adhesive technique, we presented five cases of sensors fabricated using metal electrodes and rubbers. This technique is applicable for novel cohesion between rubber and metal.

## 1. Introduction

We conducted sequential studies that led to the novel method of production of sensors made from soft rubber that was developed for artificial skin, which is expected to be required as a substitute for human or robot skin, such as hybrid skin (H-skin) [1,2,3,4,5,6,7,8,9]. Through electrolytic polymerization, rubbers with C=C bonds, such as natural rubber latex (NR-latex), are capable of cross-linking molecules adequately to be solidified in themselves. This novel solidification method is different from the usual vulcanization technique where sulfur is used at the field for the ordinary production of solid rubber. Via electrolytic polymerization, we compound a magnetic responsive liquid, i.e., magnetic compound fluid (MCF), which has nm-ordered magnetite (Fe_3_O_4_) particles that are obtained by using a magnetic fluid (MF) during compounding and has μm-ordered metal particles such as Fe, Ni, etc. By the application of a magnetic field on the compounded liquids with NR-latex and MCF during electrolytic polymerization, the magnetic particles are fabricated as heterostructures as many thin-rod shaped clusters that can be observed in organic thin film solar cells. Therefore, the electric and photovoltaic properties are enhanced, and the mechanical properties become anisotropic as well. Therefore, we named the electrolytically polymerized MCF rubber as H-Skin [7,8,9]. By doping the MCF rubber using any dopant, the electric and photovoltaic properties change as shown in Table A1 of the Appendix A. As diene-based rubbers involving isoprene rubber (IR), chloroprene rubber (CR), butadiene rubber (BR), nitrile rubber (NBR) or styrene-butadiene rubber (SBR) also have C=C bonds, they can be electrolytically polymerized. However, their Mooney viscosity must be so small such that they may be easily compounded into the MCF by adjusting the concentration of the rubber molecules. In the present study, we use NR-latex and CR-latex.

Our previous investigations have focused on the body of the sensor, but not on the fabrication of electrodes attached to the rubber. The effect of the electrode fabrication on the characteristics of the electrolytically polymerized MCF rubber should be investigated. In general, the contact resistance between the electrodes and rubber is considerably large to become an issue [10]. Moreover, with respect to the MCF rubber, the contact resistance must be reduced such that the adhesion between the electrodes and MCF rubber has higher sensitivity. This problem is related to the general research themes of adhesion between the metal and rubber [11,12,13,14,15,16,17] and the prevention of corrosion of the metal surface [18,19,20,21]. Hence, various methods of adhesion between the metal and rubber have been hitherto proposed. For example, a typical technique is the development of rubber or adhesive, involving conductive adhesives [22,23].

On the other hand, dimethylpolysiloxane (PDMS) is one of conventional soft material. There have being investigations on the bonding between PDMS and metallic or non-metallic material related to patterning with gold for a soft sensor [24]. PDMS is suitable for micro-fluidic system applications and because of transparency. PDMS generally takes on silicone oil with liquid state and the silicone oil rubber is produced with using resin to PDMS. As the silicone oil rubber is one of non-diene-based rubber without double bond C=C, PDMS may be a conventional material to produce a non-diene rubber. In contrast, the diene-based rubber involving NR, CR et al. can have many hybrid properties by conductive, photovoltaic et al. effects because it has double bond C=C. The use of the diene-based rubber such as the present MCF rubber is suitable for the purpose of rendering the rubber sensory. Therefore, in the present report, we investigate the bonding between diene rubber and metallic material.

In the present study, we propose a novel method for adhesion between the electrodes and MCF rubber by using metallic or non-metallic hydrous oxide. We investigate the mechanism of the adhesion and show the fabrication of the sensor using MCF rubber and metal electrodes.

## 2. Adhesion of Rubber and Metal

### 2.1. Adhesion between MCF Rubbers

Firstly, we investigate the adhesive effect of MCF rubber liquid on solid MCF rubber, which is useful for the fabrication of the sensor using MCF rubber and metal electrodes as described in the next section.

**Figure A1:**
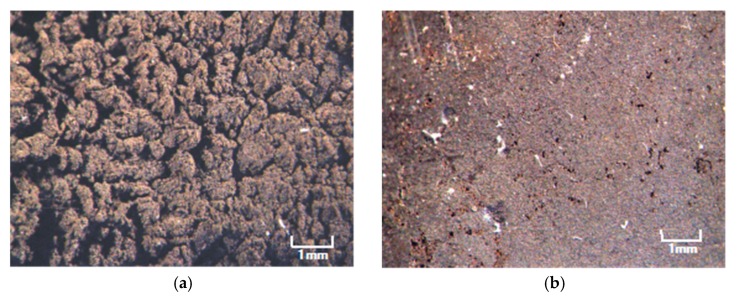
Photographs of the surface at the cathode indicated as (**a**) and anode (**b**) sides of the MCF rubber [1].

In general, the electrolytically polymerized MCF rubber surfaces are facets of the cathode and anode electrodes, shown as red and green in the figure, respectively, which is shown in detail in Figure A1 of the Appendix A. Consequently, anionic polymerization occurs at the cathode and cationic polymerization at the anode. Our used MCF rubber liquid consisted of 3 g carbonyl Ni powder, with particles of the order of µm with bumps on the surface (No. 123, Yamaishi Co., Ltd., Noda, Japan), 0.75 g water-based MF with 40 wt.% Fe_3_O_4_ (W-40, Ichinen-Chemicals Co., Ltd., Shibaura, Japan), and 3 g of NR-latex (Rejitex Co., Ltd., Atsugi, Japan). At first, the liquid was poured between stainless steel plates with a 1 mm gap; a constant electric field was applied at 6 V, an electric current of 2.7 A was passed between the plates for 10 min, and a 312 mT magnetic field was applied by permanent magnets as paired opposites across the liquid. 

**Figure 1:**
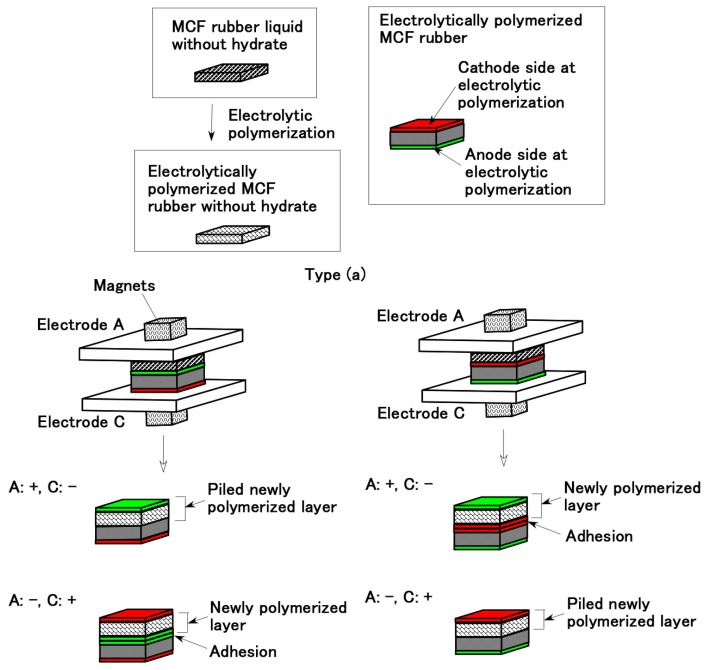
Adhesion of electrolytically polymerized in succession MCF rubber liquid without hydrate on the beforehand electrolytically polymerized solid MCF rubber without hydrate (Type (a)).

Figure 1 shows the fabrication of electrolytically in succession polymerized MCF rubber which was beforehand electrolytically polymerized. The same MCF rubber liquid was poured on the electrolytically polymerized MCF rubber surface, and a constant voltage and electric current with same intensity were applied to the same plates (see electrodes A and C in Figure 1) and magnets again. Through this second electrolytic polymerization, the electrolytically polymerized layer is created anew, which is independent of the beforehand electrolytically polymerized MCF rubber surfaces and the electric poles of the electrodes.

**Figure 2:**
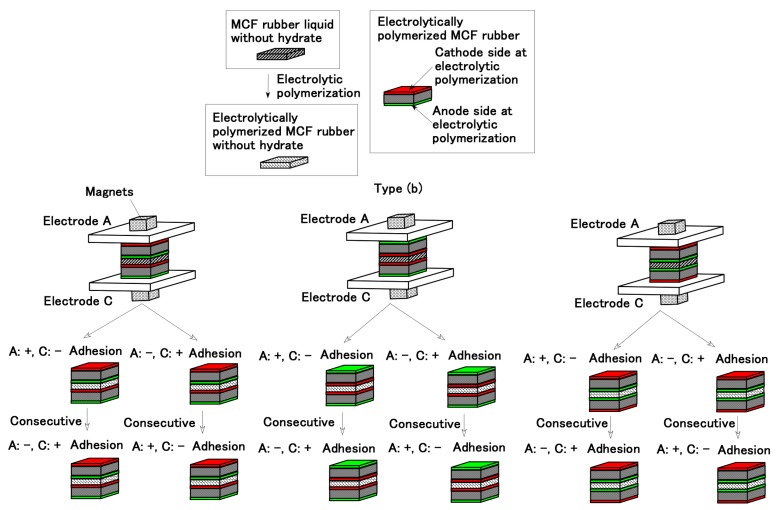
Adhesion between beforehand electrolytically polymerized MCF rubbers without a hydrate electrolytically polymerized again by MCF rubber liquid without a hydrate (Type (b)).

Next, we investigate whether the MCF rubber liquid can be used to bond the two beforehand electrolytically polymerized MCF rubbers as shown in Figure 2. The constituent of the MCF rubber and conditions of electrolytic polymerization are the same that the ones in Figure 1, where the electrolytic polymerization is conducted twice by replacing the electric poles of the electrodes, as shown by “Consecutive” in the figure. Regardless of the cathode and anode electrodes sides during the electrolytic polymerization of the beforehand electrolytically polymerized MCF rubber surfaces and electric poles of the electrodes, the MCF rubber liquid can adhere anew to bond between the beforehand electrolytically polymerized MCF rubbers.

From the results of Types (a) and (b) for Figure 1 and Figure 2, the adhesion on the surface of the beforehand electrolytically polymerized MCF rubber by MCF rubber liquid is independent of the cathode and anode electrodes sides during the electrolytic polymerization of the beforehand electrolytically polymerized MCF rubber surfaces.

### 2.2. Adhesion between MCF Rubber and Metal

**Figure 3:**
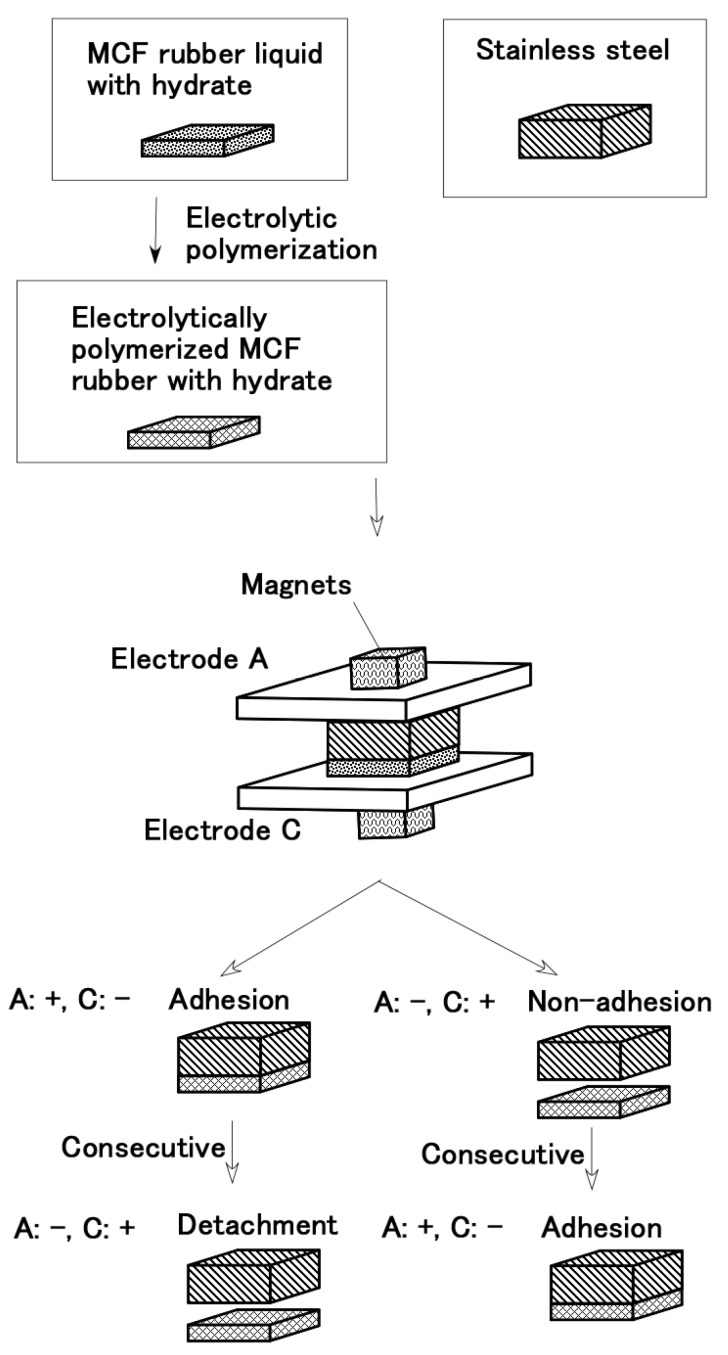
Adhesion between metal and MCF rubber liquid with the hydrate.

**Figure A2:**
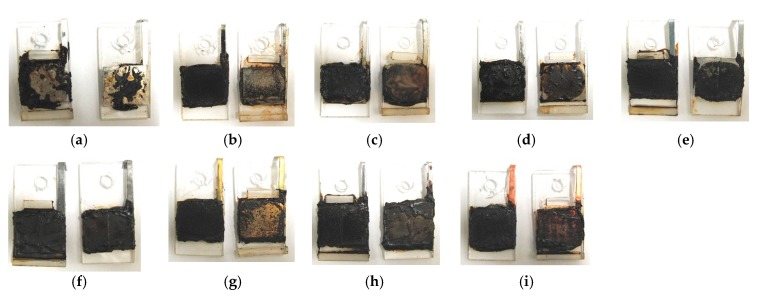
Photographs of each anode and cathode metal electrode surface (20 mm × 22 mm surface size) separated into parts from facing state each other after the electrolytic polymerization: the black as seen in the photographs is electrolytically polymerized MCF rubber affixed on the electrode; the anode is on the left side and the cathode is on the right side; (**a**) aluminum; (**b**) stainless steel; (**c**) titanium; (**d**) nickel; (**e**) zinc; (**f**) lead; (**g**) brass; (**h**) iron; (**i**) copper.

In this section, we investigate the adhesion between MCF rubber and metals. In general, the MCF rubber cannot be adhered on any metal as shown by Figure A2 in the Appendix A. Therefore, we must develop the MCF rubber liquid using certain methods. We attempted to compound metallic hydrous oxide into the MCF rubber liquid: sodium tungstate (VI) dehydrate, Na_2_WO_4_·2H_2_O (Fujifilm Wako Pure Chemical Co., Ltd., Osaka, Japan); disodium molybdate (VI) dehydrate, Na_2_MoO_4_·2H_2_O (Fujifilm Wako Pure Chemical Co., Ltd., Osaka, Japan). In addition, we also attempted to compound non-metallic hydrous oxide: sodium tetraborate decahydrate, Na_2_B_4_O_7_·10H_2_O (Fujifilm Wako Pure Chemical Co., Ltd., Osaka, Japan). Metallic hydrous oxide is a metal complex formed by the crystallization of metal oxide and H_2_O in radical condition. Non-metallic hydrous oxide is also fabricated by crystallization and involving H_2_O to be in radical condition. The MCF rubber liquid consisted of 3 g carbonyl Ni powder, 0.75 g water-based MF (W-40), 3 g NR-latex, and 0.75 g hydrate (as for each above designated hydrous oxide). During the first stage of the production of the MCF rubber liquid with a hydrate, hydrate and MF should be mixed slowly by using a supersonic vibrator (UR-20P, Tomy Seiko Co. Ltd., Tokyo, Japan) for resolution such that H_2_O in the hydrate percolates through the MF. The liquid was poured on stainless steel plates and a constant electric field was applied at 6 V between the stainless steel plates A and C with a 1 mm gap, and an electric current of 2.7 A was passed between the plates for 10 min and a 312 mT magnetic field was applied by permanent magnets as paired opposites across the liquid, as shown in Figure 3. 

We selected stainless steel as the electrode material for the purpose of reducing corrosion and smooth detachment from the electrodes A and C. During electrolytic polymerization, the metal must be on the anode side for the MCF rubber liquid with the hydrate to adhere to the metal. If it is on the cathode side, they become detached, as seen from “Consecutive” in Figure 3. 

Furthermore, we investigate the cause of the results of Figure 3. Now, we consider the hydrate Na_2_WO_4_·2H_2_O. W is coordinated with surrounding O at the center of crystal structure by crystallization. At the anode electrode side, the hydrate is ionized in the compounded MCF and the ion WO_4_^2−^ is a radical as shown in Equation (1). The electrons in the third term of Equation (1) percolate through the anode.

(1) $$Na_{2}WO_{4}\cdot 2H_{2}O\to 2Na^{+}+:WO_{4}+2e^{-}+2H_{2}O$$

**:**WO_4_ is radically reacted divalent state with the isoprene molecule of NR-latex as shown by Equation (2), where **:**WO_4_ is replaced to **:**Br and isoprene molecule to RH, and that R is rewritten as shown by Equation (3).

(2) $$:Br+RH\to \cdot Br\overset{•}{R}H+\cdot BrR+H^{•}$$

(3)
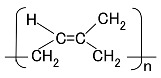


**Figure 4:**
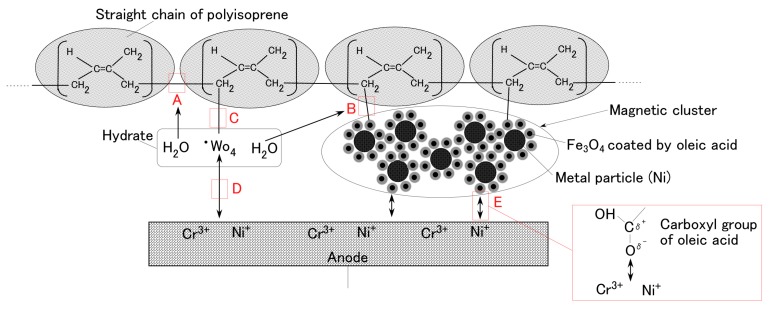
Schematic model of electrolytic polymerization of MCF rubber liquid with the hydrate.

**^·^**BrR is presented as Equation (4): the hydrate and isoprene molecule are cross-linked as shown by C in Figure 4. Figure 4 is the schematic model of electrolytic polymerization of MCF rubber liquid with the hydrate. In addition, **^·^**BrR is ionized anionic so that it is adhered with the stainless steel metal of the anode which is SUS304 and is ionized cationic by Ni^+^ and Cr^+^ as shown by D in Figure 4, where Ni^+^ is occurs by Equation (5) because the ionization tendency of Ni of stainless steel is a little larger than that of H in the MCF rubber liquid.
(4)
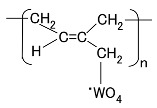

(5) $$Ni+2H^{+}\to Ni^{+}+H_{2}$$

On the other hand, H_2_O in the right hand of Equation (1) contributes to the electrolytic polymerization of isoprene molecule and oleic acid which is coated around Fe_3_O_4_ particle as shown by A and B in Figure 4, whose mechanism has been presented in the previous study [1,5]. The oleic acid molecule coated around Fe_3_O_4_ particles is ionized by the mesomeric effect [25] as shown by Equation (A1) in Appendix A and by “E” in Figure 4.

**Figure 5:**
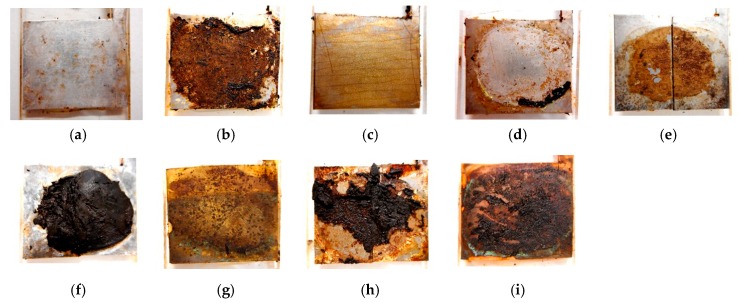
Photographs of anode metal electrode surface adhered by MCF rubber with the hydrate Na_2_WO_4_·2H_2_O separated into parts from facing cathode each other after the electrolytic polymerization: electrolytic polymerization with 1 mm metal plates gap, constant electric field at 6 V, 2.7 A and 10 min, 312 mT magnetic field generated by permanent magnets as paired opposites; metal plate has 20 mm × 22 mm surface size; 1 g carbonyl Ni powder, 0.75 g water-based MF (W-40), 3 g NR-latex, and 0.5 g hydrate; (**a**) aluminum; (**b**) stainless steel; (**c**) titanium; (**d**) nickel; (**e**) zinc; (**f**) lead; (**g**) brass; (**h**) iron; (**i**) copper.

**Figure 6:**
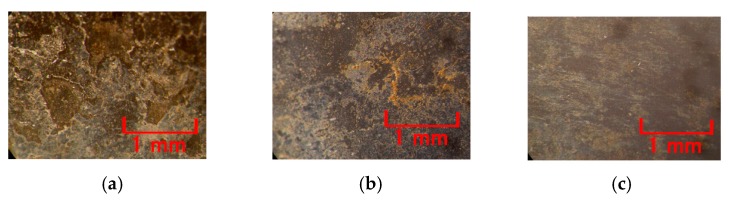
Photographs of the anode electrode surface to be detached from the electrolytically polymerized MCF rubber with the hydrate: with 1 mm metal plates gap, constant electric field at 6 V, 2.7 A and 10 min, 312 mT magnetic field by permanent magnets as paired opposites; 1 g carbonyl Ni powder, 0.75 g water-based MF (W-40), 3 g NR-latex, and 0.5 g hydrate; (**a**) Na_2_WO_4_·2H_2_O; (**b**) Na_2_MoO_4_·2H_2_O; (**c**) Na_2_B_4_O_7_·10H_2_O.

The factors that contribute to the adhesion between the MCF rubber with the hydrate and the metal electrode are the ionic bond between radical **^·^**WO_4_ and cationic metal electrode as shown by "D" in Figure 4 and that between anionized oleic acid molecule and cationic metal electrode as shown by "E" in Figure 4. The adhesion effect by the former becomes larger than that of the latter, as the intensity of the work function of the metal molecule of hydrate such as W^6+^ or Mo^6+^ is at the same level of the work function of metal molecule of the electrode such as Cr and Ni. From the following results shown in Figure 5, however, the factor that contributes towards the ionic bond in the hydrate is not Ni but Cr. As for B^3+^, the work function is enough to be considered by replacing to redox potentials. Therefore, because of the work function and redox potentials, the metal surface is reacted electrochemically: the corrosion in the case of Na_2_WO_4_·2H_2_O and Na_2_MoO_4_·2H_2_O is large and the one in the case of Na_2_B_4_O_7_·10H_2_O is small, as shown by Figure 6.

Each case of Na_2_MoO_4_·2H_2_O and Na_2_B_4_O_7_·10H_2_O, WO_4_^2−^ is replaced to MoO_4_^2−^ and B_4_O_7_^2−^ respectively in Equations (1)−(4) such that the same adhesion mechanism can be considered. From this mechanism, the adhesion factor is a hydrate with metallic or non-metallic element with more than two valency. In contrast, from another test, it was verified that the hydrates with chloride ion Cl^−^, hydroxide ion OH^−^, citric acid ion C_6_H_5_O_7_^3−^, phosphoric acid PO_4_^3−^, nitric acid ion NO_3_^−^, sulfuric acid SO_4_^2−^, and ammonium ion NH_4_^+^ cannot contribute towards the adhesion effect. As shown in Figure 6, the metals which can be adhered to the MCF rubber with the hydrate are stainless steel, iron, and lead; those that cannot be adhered are aluminum, titanium, nickel, zinc, brass, and copper. The case of lead has larger adhesion effect than that of stainless steel. Incidentally, as the present novel adhesion technique is applicable to rubber-type solar cells, in the case of the transparent electrode, the electrode without TiO_2_ coating cannot be adhered but the one with TiO_2_ coating can be adhered. The reason behind the adhesion is the difference of intensity of the work function or redox potentials between the electrode’s metal and metal molecule of the hydrate.

In conclusion, MCF rubber with the hydrate has the role of adhesive bonding with metal. As shown in Figure 1, Figure 2, Figure 3, Figure 5 and Figure 6 the reason for using a magnet is to make the MCF rubber exhibit anisotropy electrically and mechanically, and to have the MCF rubber liquid aggregated such that the electrolytic polymerization enhances.

Compared to NR-latex, diene-based rubber is more practical for diverse engineering applications as it can withstand various ambiences to a greater extent. As the diene-based rubber has C=C bonds, it is feasible to be electrolytically polymerized. Therefore, the anionic metal of hydrate and CR are also cross-linked as shown in Equation (4). Then, we dealt with CR-latex and compounded it into the NR-latex-based MCF rubber liquid. Namely, the MCF rubber liquid consisted of 3 g Ni powder, 0.75 g MF (W-40), 3 g NR-latex, and 3g CR-latex (671A, Showa Denko Co. Ltd., Tokyo, Japan) for Types (a) and (b) in Figure 1 and Figure 2, and consisted of 1 g Ni powder, 0.75 g MF (W-40), 3 g NR-latex, 3g CR-latex (671A) and 0.5 g hydrates for Figure 3, Figure 5 and Figure 6. In the case of compounding CR-latex to NR-latex-based MCF, we can obtain the same results of Figure 1, Figure 2, Figure 3, Figure 5 and Figure 6.

**Figure 7:**
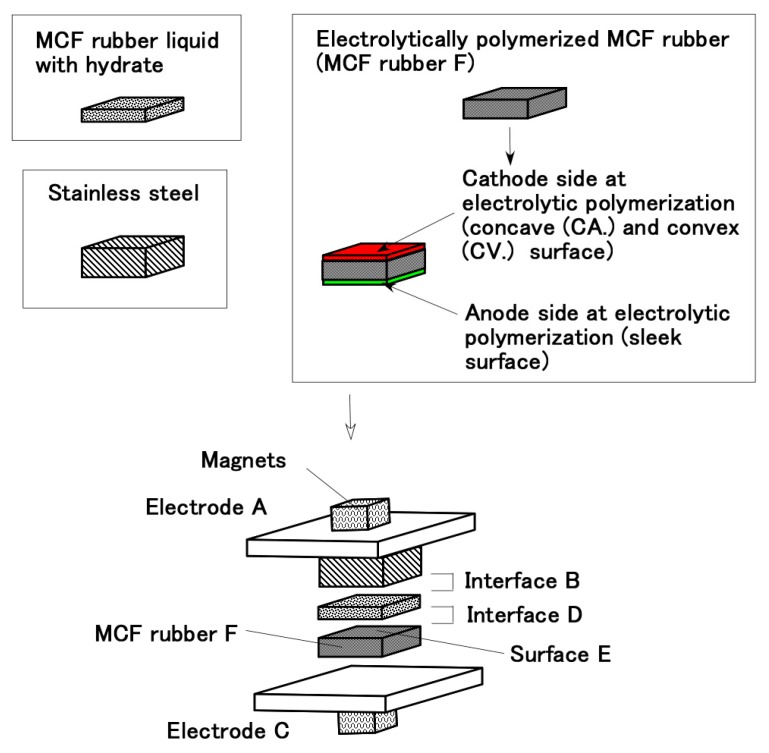
Fabricate of the beforehand electrolytically polymerized MCF rubber F, MCF rubber liquid involving CR-latex and NR-latex with the hydrate and stainless steel.

As shown in Figure 7, therefore, we fabricated the beforehand electrolytically polymerized MCF rubber F, MCF rubber liquid involving CR-latex and NR-latex with the hydrate as adhesive, and stainless steel as the electrode of the sensor. After we electrolytically polymerized the structure we investigated whether interfaces B and D can be adhered or not. The MCF rubber liquid involving CR-latex and NR-latex with the hydrate consisted of 1 g Ni powder, 0.75 g MF (W-40), 3 g NR-latex, 3 g CR-latex (671A) and 0.5 g Na_2_WO_4_·2H_2_O. The solid MCF rubber F in Types (A)−(D) are shown in Table 1.

The results of interfaces B and D are shown in Table 2 and Table 3. The former represents the cohesion between MCF rubber F and MCF rubber liquid with the hydrate, the latter represents the cohesion between metal and MCF rubber liquid with the hydrate. Electrolytic polymerization was consecutively conducted on again by reversing electrodes A and C. Regardless of the types of MCF rubber F, the surface E with CA. or CV., i.e., electrode poles of A and C, and consecutive reversing electrode poles of A and C, interface D becomes adhesion A, which is the same tendency as observed in the Types (a) and (b) in Figure 1 and Figure 2. On the other hand, interface B exhibits A. or N. according to variegated conditions of the types of MCF rubber F, the surface E with CA. or CV., and the electrode poles of A and C.

**Figure 8:**
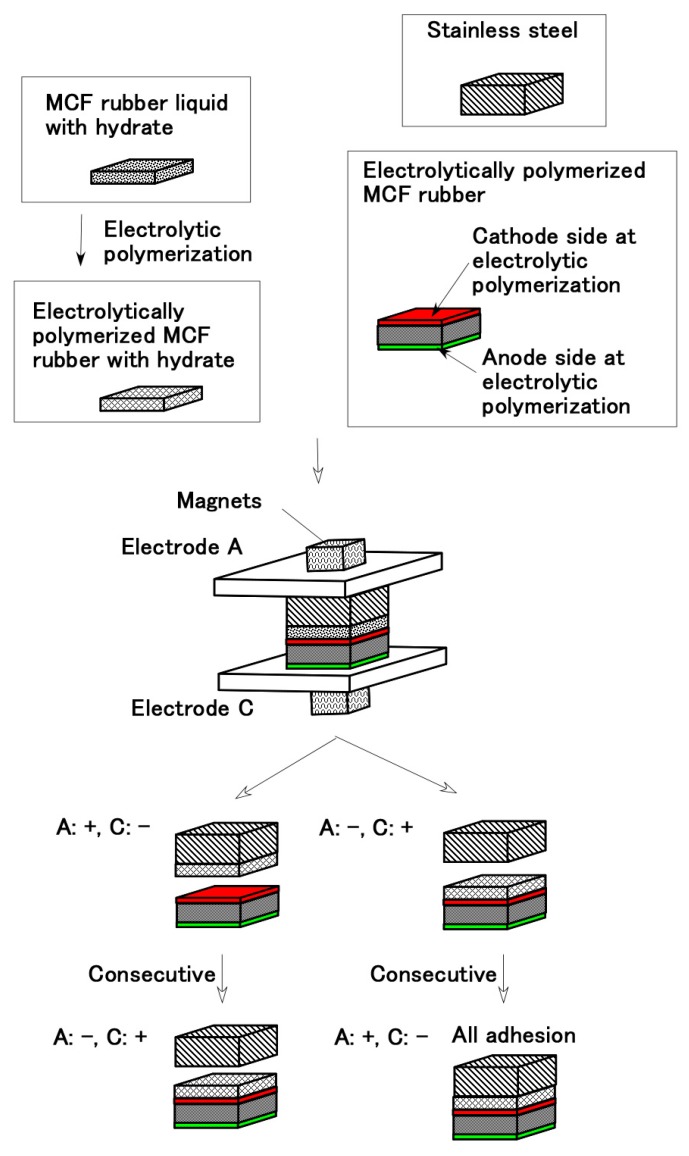
Production of adhesion of beforehand electrolytically polymerized MCF rubber and metal, which is concluded from the results of Table 2 and Table 3.

Referring to (b) in Table 2 and Table 3, however, a conclusive result of sensor production can be obtained as shown by Figure 8 in the case of the MCF rubber F including CR-latex. In contrast, regardless of MCF rubber F’s including CR-latex or not, interfaces B and D can have adhesion A as another sensor production by the condition as shown by (a) in Table 2 and Table 3. The difference in electric properties between by (a) and (b) in Table 2 and Table 3 will be argued in the following section. From Figure 8, the bonding process by the present technique is summarized to be categorized simply as follows.

1. When the pole of metal at electrolytic polymerization is anode, the rubber with hydrate can be adhered to the metal. (Incidentally, when the pole of metal at electrolytic polymerization is reversed cathode consecutively, the rubber with hydrate is detached from the metal.)
2. When the pole of beforehand electrolytically polymerized MCF rubber (called for convenience “bep-MCF rubber”) at electrolytic polymerization is anode, the rubber with hydrate can be adhered to the bep-MCF rubber. (In addition, the bep-MCF rubber’s surface poured by the rubber with hydrate is preferable cathode side at beforehand electrolytic polymerization.)
3. At first, the rubber with hydrate should be adhered to the bep-MCF rubber by making the bep-MCF rubber anode, and then the rubber with hydrate should be adhered to the metal by making the metal anode.

## 3. Fabrication of Sensor

**Figure 9:**
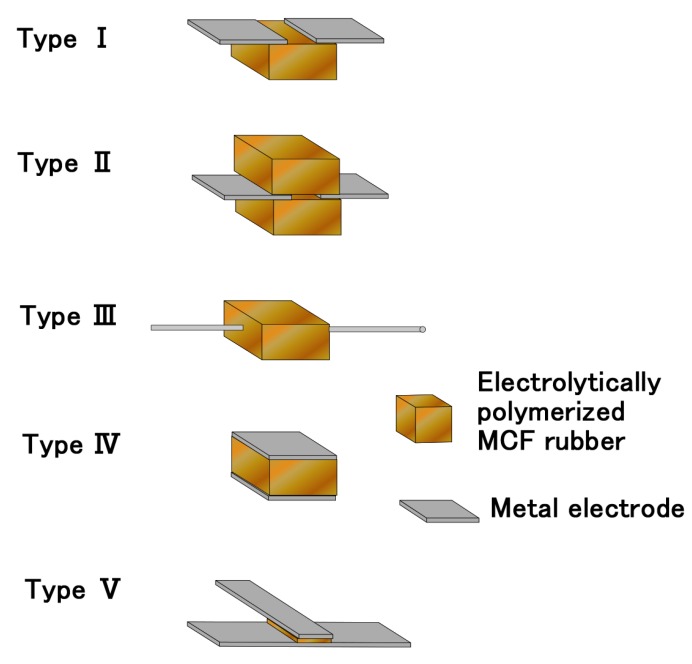
Various types of sensors fabricated by the adhesion of electrodes to MCF rubber from the results of previous sections.

Based on the results of Figure 8, we can propose five types of sensors fabricated by the adhesion of electrodes to MCF rubber as shown in Figure 9. Types I–III are applicable for the field to be requested in rubber’s stretching motion or in sensing electric property at shear motion such that the sensor’s motion is parallel to shear force. In particular, type III is appropriate for artificial skin installed on a robot such as the H-Skin that we have proposed such that electrodes are fine lines and do not interfere with the skin’s dynamic motion. Type IV is applicable for sensor such as piezo element and is appropriate to sense electric property under pressure applied perpendicularly to the electrode. This type is not suitable for sensor’s shear motion because the touching surface of the sensor is not a soft rubber, rather a solid electrode. Type V is applicable for joining between electrodes for sensors.

Basic principle is MCF rubber liquid with hydrate is adhesive bonding between MCF rubber without hydrate and metal electrode.

**Figure 10:**
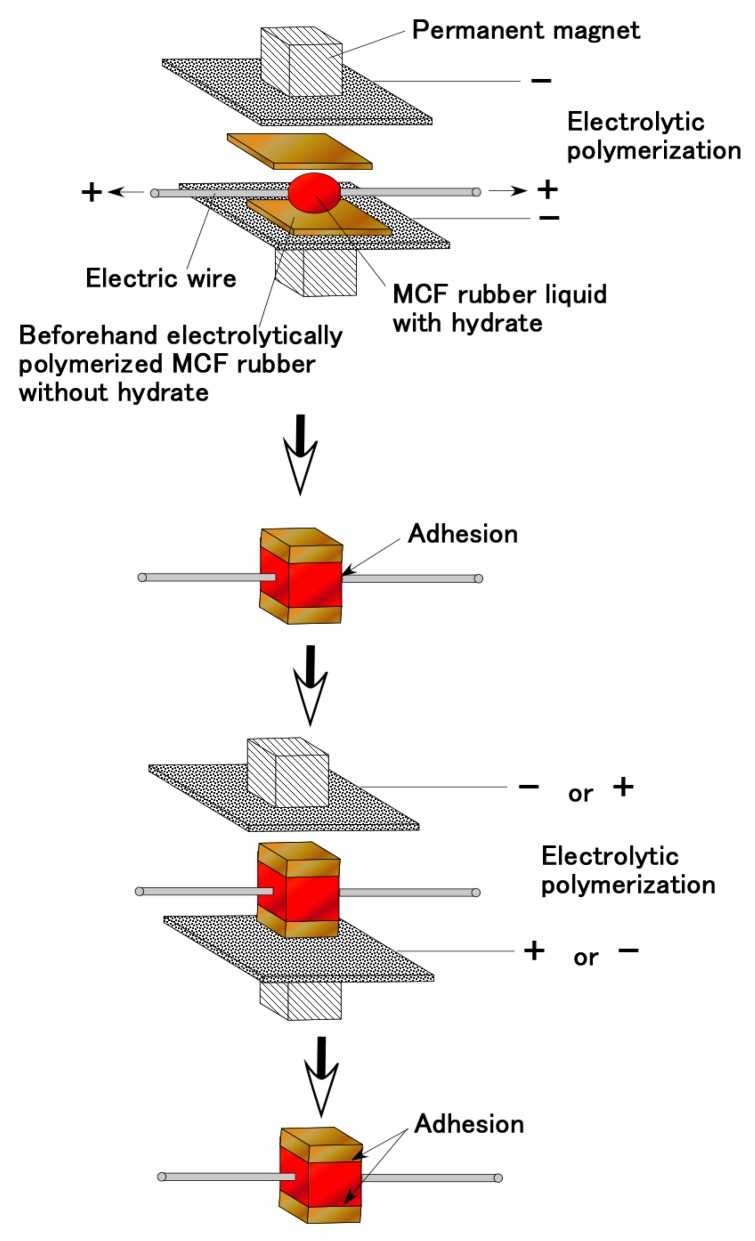
Production procedure as for type III in Figure 9.

**Figure 11:**
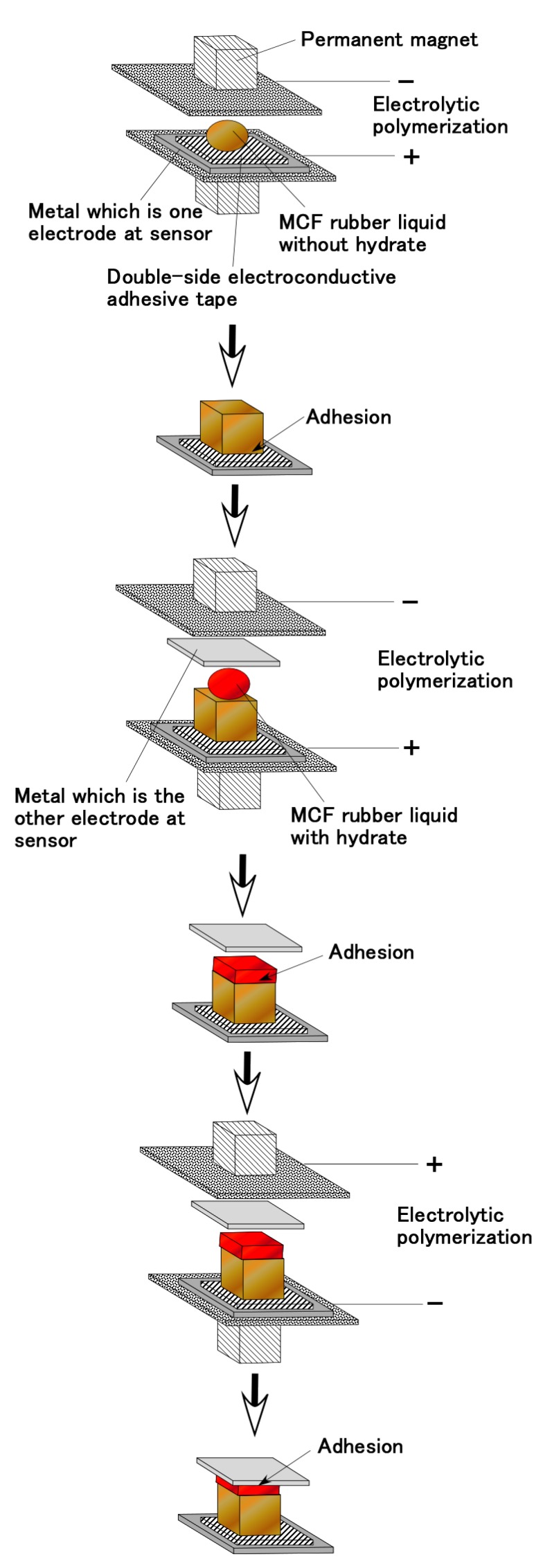
Production procedure for type IV sensor shown in Figure 9.

**Figure 12:**
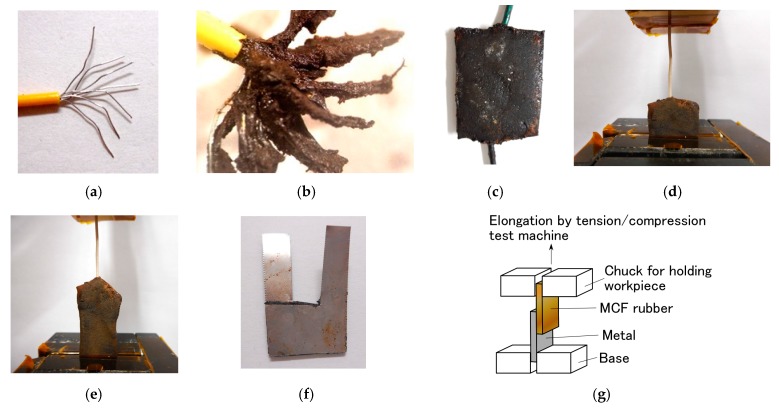
Photographs of type III and IV sensors shown in Figure 9: (**a**) electric wire as electrodes of type III sensors; (**b**) thin electric wires affixed electrolytically polymerized MCF rubber with the hydrate; (**c**) completed MCF rubber sensor of type III; (**d**) completed MCF rubber sensor of type III installed at a tension/compression test machine; (**e**) completed MCF rubber sensor of type III elongated by a tension/compression test machine; (**f**) completed MCF rubber sensor of type IV; (**g**) rubber of completed MCF rubber sensor of type IV elongated by a tension/compression test machine.

As for Types III and IV, the production procedure is shown in Figure 10 and Figure 11, respectively. Regarding Figure 10, at first, MCF rubber liquid with hydrate inserted by two electric wires with several thin wires as shown in Figure 12a is sandwiched between two beforehand electrolytically polymerized solid MCF rubbers, and then a constant voltage and electric current are supplied under the wires as anode and the solid MCF rubbers as cathode such that the wires are adhered to the MCF rubber with hydrate. Next, a constant voltage and electric current are supplied under the solid MCF rubbers as cathode or anode such that the solid MCF rubbers are adhered to the MCF rubber with hydrate. The completed sensor is shown in Figure 12c. Where the wire as shown in Figure 12a has about φ1.3 mm outer diameter with seven thin silver-gilt electric wires with about φ0.1 mm diameter and about 5 mm length. Although the number of thin silver-gilt wires is less and their diameter is very small, the wires cannot be detached through the electrolytically polymerized MCF rubber affixed around each thin wire as shown in Figure 12b. Therefore, the wire electrodes cannot be detached by elongation as shown in Figure 12e under settlement of the MCF rubber sensor at a commercial, small-size tension/compression test machine (SL-6002, IMADA-SS Co. Ltd., Toyohasi, Japan) as shown in Figure 12d at initial settlement figure. 

Regarding Figure 11, at first, MCF rubber liquid without hydrate is poured on a metal affixed by double electroconductive adhesive tape which should be one electrode of sensor, and then a constant voltage and electric current are supplied under the metal as anode such that the metal is adhered to the MCF rubber. The cause of using the adhesive tape is that both metals as electrodes of sensor cannot be adhered simultaneously to the MCF rubber with hydrate by reversing the electric poles many times as follows because of opposite position of the two metals if not use the adhesive tape. In general, the adhesive tape includes NR-latex, therefore, by electrolytic polymerization MCF rubber liquid can be piled on the adhesive tape. Next, MCF rubber liquid with hydrate is poured on the electrolytically polymerized solid MCF rubber without hydrate (the poured surface of the solid MCF rubber is concave and convex surface is more appropriate for adhesion), and another metal, which should be the other electrode of the sensor, is placed on the MCF rubber liquid with hydrate. In addition, then a constant voltage and electric current are supplied under the solid MCF rubber as anode and another metal as cathode such that the solid MCF rubber is adhered to the MCF rubber with hydrate. Lastly, the electric poles of electrolytic polymerization are reversed, and then a constant voltage and electric current are supplied again such that another metal is adhered to the MCF rubber with hydrate. The completed sensor is shown in Figure 12f. Where metal is stainless steel with 0.1 mm thickness.

The completed sensor as shown in Figure 12c,f was consisted of MCF rubber liquid with hydrate as 1 g Ni powder, 0.75 g MF (W-40), 3 g NR-latex, 3g CR-latex (671A), and 0.5 g hydrates, and MCF rubber without hydrate as 3 g Ni powder, 0.75 g MF (W-40), 3 g NR-latex, and 3g CR-latex (671A) with 1 mm metal plates gap, constant electric field at 6 V, 2.7 A and 5 min under atmosphere, 312 mT magnetic field by permanent magnets as paired opposites at each electrolytic polymerization.

**Figure 13:**
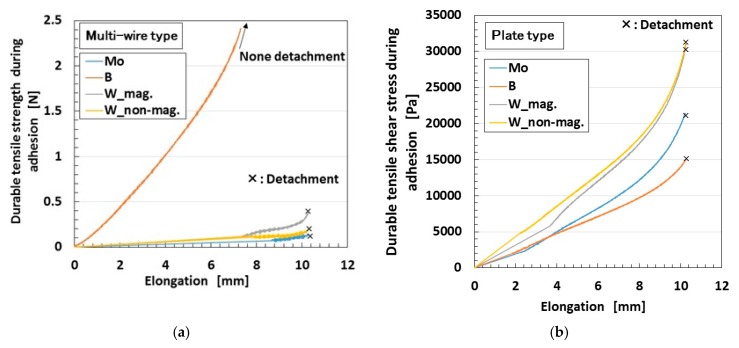
Durable tensile force during adhesion upon elongation: (**a**) for type III sensor; (**b**) type IV sensor: each electrolytic polymerization with 1 mm metal plates gap, constant electric field at 6 V, 2.7 A and 5 min with a 312 mT magnetic field applied using permanent magnets as paired opposites; the MCF rubber liquid with a hydrate is composed of 1 g carbonyl Ni powder, 0.75 g water-based MF (W-40), 3 g NR-latex, 3 g CR-latex (671A), and 0.5 g hydrate; MCF rubber without the hydrate is composed of 3 g carbonyl Ni powder, 0.75 g water-based MF (W-40), 3 g NR-latex, and 3 g CR-latex (671A); “mag.” or “non-mag.” means with or without the use of magnets at electrolytic polymerization, respectively.

Using the tension/compression test machine (SL-6002), we investigated the resistant force of electrodes for detachment from the sensor for the completed sensors of Types III and IV as shown in Figure 13a, b, respectively. As for type III, when one side of electric wires was elongated by fixing a part of the rubber as shown in Figure 12d, durable tensile force during adhesion was measured. As for type IV, when the electrolytically polymerized MCF rubber without the hydrate was adhered to 0.1 mm-thick stainless steel using the MCF rubber liquid with the hydrate, and was elongated by fixing the stainless steel on the base of the test machine as shown in Figure 12g, durable tensile force during adhesion was measured. The contact area between stainless steel and MCF rubber is about 12 mm × 15 mm. Cross marks in the figures indicate the detachment of the wire from the sensor. As for multi-wire type shown in Figure 13a, because the silver-gilt wires are very thin and can be made with the same length, the durable tensile force is appropriate for evaluation by strength rather than by pressure, which is different from the arrangement in the case of the plate type in Figure 13b. The used hydrates are Na_2_WO_4_·2H_2_O (indicated as “W” in the figure), Na_2_MoO_4_·2H_2_O (“Mo”,) and Na_2_B_4_O_7_·10H_2_O (“B”). Figure 13 also shows the case of Na_2_WO_4_·2H_2_O, i.e., the case without a magnet during the production of the sensor.

As for multi-wire-type sensor, the detachment in the case of Na_2_B_4_O_7_·10H_2_O is not presented because the durable tensile strength is very large. Experimental results confirmed that this type of sensor is durable up to about 10 N, which corresponds to 1 kg weight loaded on the electric wire of the sensor, as shown in Figure 12e.

In the case of multi-wire type, the durable tensile strength in the case of Na_2_B_4_O_7_·10H_2_O is the highest. However, in the case of plate type, the highest value sis obtained for Na_2_WO_4_·2H_2_O. The cause can be hypothesized as follows. The area of electrolytic polymerization at the multi-wire type is smaller than that at the plate type. The difference in the electrolytic polymerization is due to the different kinds of hydrate. 

On the other hand, no effect of magnetic field during electrolytic polymerization can be seen. However, the using of magnets is important to collect the MCF rubber liquid by using the magnetic field during sensor production.

**Figure 14:**
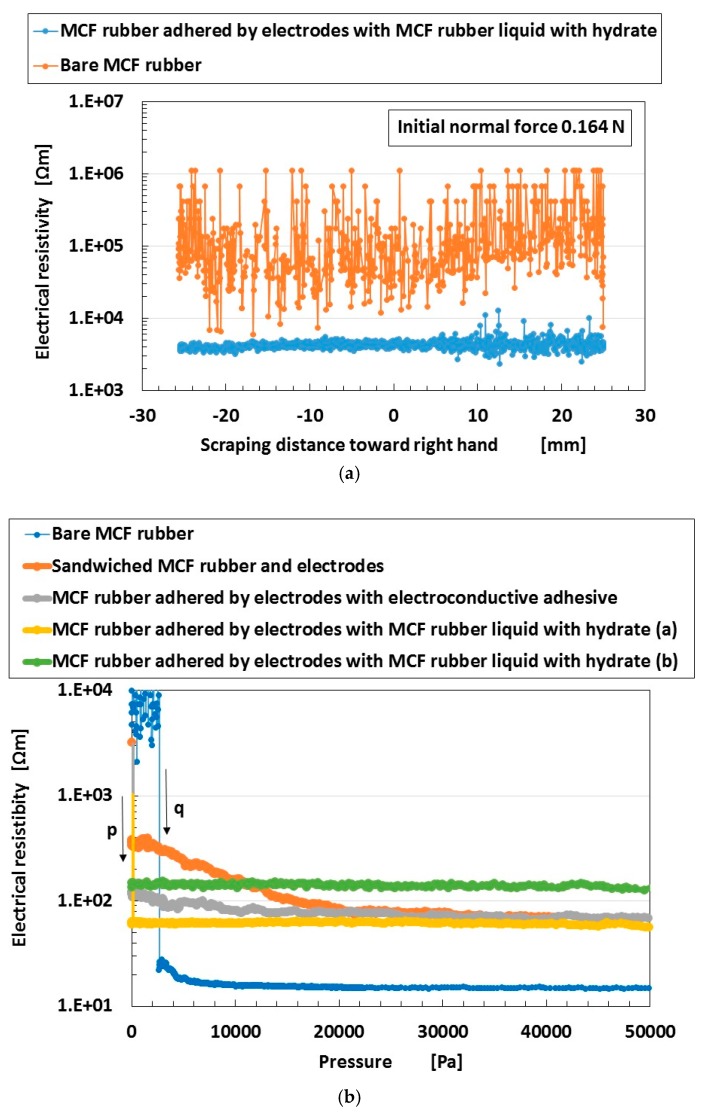
Electric characteristics of completed MCF rubber sensor: (**a**) for type III sensor; (**b**) type IV sensor: electrolytic polymerization with 1 mm metal plates gap, constant electric field at 6 V, 2.7 A and 5 min and 312 mT magnetic field applied by permanent magnets as paired opposites; the MCF rubber liquid with the hydrate is composed of 1 g carbonyl Ni powder, 0.75 g water-based MF (W-40), 3 g NR-latex, 3 g CR-latex (671A), and 0.5 g hydrate; MCF rubber without the hydrate is composed of 3 g carbonyl Ni powder, 0.75 g water-based MF (W-40), 3 g NR-latex, and 3 g CR-latex (671A).

Finally, we investigate the electric characteristics of completed Types III and IV sensors. The former is appropriate for sensing in shear motion. Therefore, we used the experimental apparatus for the measurement of electrical resistivity of the MCF rubber that is moving at 0.164-N normal force and 5-mm/s sweeping velocity to scrape a body with some surface roughness, which was used in the previous study [1]. The voltage 10 V was supplied between the two electric wires by connecting the electric resistance of 1.8 kΩ. The MCF rubber sensor was made to rub a flat plate with a surface roughness of *R_a_* = 20.86 μm, *R_y_* = 199.9 μm, and *R_q_* = 26.89 μm. The MCF rubber sensor was moved parallel to the material surface by an actuator with a constant speed and 50 mm scraping distance under a normal force which is presented as the one during the initial movement in the figure. A hard, non-electric body with φ0.5 mm diameter was interposed between the MCF rubber sensor and the acrylic resin body so that the MCF rubber sensor could be contacted exactly. The experimental procedure is referred to as the shear force experiment (SFE) [1]. The result is shown in Figure 14a, with a comparison with results for bare electrolytically polymerized MCF rubber without a hydrate, which means that the MCF rubber is not fabricated as type III sensor and is a sole MCF rubber indicated as “beforehand electrolytically polymerized MCF rubber without hydrate” delineated in Figure 10. The electrical resistivity of completed sensor is more stable without perturbation than that of bare MCF rubber.

Furthermore, the type IV sensor is appropriate for sensing during pressing. Therefore, we used the experimental apparatus for measuring the electrical resistivity of the MCF rubber that is pressed by applying a normal force, which was used in the previous study [1]. The voltage 10 V was supplied between the two electric wires by connecting the electric resistance of 1.8 kΩ. Using a tension/compression test machine (SL-6002), the MCF rubber was placed between two bodies, i.e., 7-mm square stainless steel plates. The upper body was moved to touch the lower one by an actuator at a pressing speed of 10 mm/min. The experimental procedure is referred to as the normal force experiment (NFE) [1]. The result is shown in Figure 14b, and compared with the results obtained when using the bare electrolytically polymerized MCF rubber without the hydrate, which means that the MCF rubber is the same that the bare MCF rubber in Figure 14a, and of various types of MCF rubber sensors. The first MCF rubber sensor is the MCF rubber just sandwiched by two stainless steel plates as electrodes without any adhesives (which is named “MCF rubber sensor 1” here). The second one is the MCF rubber sandwiched to be adhered to two stainless steels as electrodes with double electroconductive adhesive tape (which is named “MCF rubber sensor 2” here). The third and the fourth ones are MCF rubber sensor made by the production procedures (a) and (b) in Table 2 and Table 3, respectively. The latter corresponds to type IV made as shown in Figure 11 (which is named “MCF rubber sensor (b)” here), and the former is the simple case of type IV as described as follows (which is named “MCF rubber sensor (a)” here). The MCF rubber liquid without the hydrate is poured on a metal affixed using the double electroconductive adhesive tape to obtain one electrode of the sensor, and then, a constant voltage and electric current are supplied under the metal as the anode such that the metal is adhered to the MCF rubber.

Next, the MCF rubber liquid with the hydrate is poured on the electrolytically polymerized solid MCF rubber without a hydrate, and another metal that should be the other electrode of the sensor is placed on the MCF rubber liquid with the hydrate. These steps are the same as those in case (b). The next step is different. A constant voltage and electric current are supplied under the solid MCF rubber as the cathode and another metal as the anode such that the solid MCF rubber and another metal are simultaneously adhered to the MCF rubber with the hydrate.

In the case of bare MCF rubber, the initial electrical resistivity without pressure is the larger than that in case of any MCF rubber sensors; however, upon pressing, it becomes smaller by ~15 Ω m, which is smaller than those in case of any MCF rubber sensors. However, because electrodes are needed in a sensor, the bare MCF rubber does not fulfill the requirement. In the case of MCF rubber sensor 1, the electrical resistivity in the smallest pressure range is smaller than that of bare MCF rubber. In the case of MCF rubber sensor 2, the electrical resistivity in the smallest pressure range is smaller than that of MCF rubber 1. If an adhesive tape is not used, the electrodes are easily detached from the rubber sensor. Therefore, these results indicate that MCF rubber sensor 2 is superior to MCF rubber sensor 1. In the case of MCF rubber sensor (b), the initial electrical resistivity in the smallest pressure range is smaller than that of MCF rubber 2 by ~60 Ω m, which is 10-ordered Ωm the same as that of bare MCF rubber and is smaller than that of any other MCF rubber sensors. The electrical resistivity does not vary with increasing pressure. The results indicate that the sensor produced by the present adhesive production technique with the hydrate is superior to the MCF rubber sensor made without firm adhesion of electrodes to the sensor (e.g., MCF rubber sensor 2). Furthermore, they also indicate that we can achieve electric conductivity between rubber and metal smaller from the beginning of application of pressure by using the present adhesive production technique. In the case of the MCF rubber sensor (a), the electrical resistivity is larger than that of the MCF rubber sensor (b). The cause of the results is that the coordination of electric current’s passing inner the MCF rubber fabricated by particle and molecules of the MCF rubber is intensified by multiple electrolytic polymerization. Therefore, the present adhesive production technique corresponding to (b) in Table 2 and Table 3 is superior to that corresponding to (a) in Table 2 and Table 3. On the other hand, the cases of MCF rubber sensor 1, 2, (a) and (b) are abruptly changed by enhanced pressure around the initial pressure as indicated “p” in Figure 14b. This tendency has more agile switching effect by the least pressure rather than the case of bare MCF rubber as indicated “q” in Figure 14b.

As for reliability and durability of the present fabricated sensor, we suggest as follows. According to the period of electrolytic polymerization, the secular change of property of the fabricated sensor differs. The cause is due to the water involved in the fabricated sensor. Because of the use of NR or CR, water gets into the MCF rubber. The volatility differs according to the degree of electrolytic polymerization: if the period of the electrolytic polymerization is short, the amount of water imported into the structure of the MCF rubber’s molecular and particles is little, and the MCF rubber sensor is wet. Because the volatility of water occurs, the property of the MCF rubber sensor changes in time; on the contrary, if the period is long, the amount of water imported into the structure is much, and the MCF rubber sensor is arid. Because of small volatility of water, the property of the MCF rubber sensor holds constant in time. Therefore, the reliability and durability of the fabricated sensor depends on the electrolytic polymerization period. Thus, consequence is also dependent on the kind of fabrication of five sensors as shown in Figure 9, for example, type IV has feasibility of longer durability than type III. Concerning to enhancement of the durability, we can propose many methods, for example, we displace other kind of the rubber. CR is more suitable for reducing volatility of water than NR. Therefore, we used CR in the present study. On the other hand, we can prose the others and intend to report the results in other reports.

## 4. Conclusions

To produce a sensor with electrodes, we proposed a novel adhesive technique with the use of rubber with C=C bonds, such as MCF rubber, NR-latex, and CR-latex compounded with a hydrate by electrolytic polymerization under a magnetic field. The recommendable hydrate is a metallic or non-metallic element with more than two valency, i.e., Na_2_WO_4_·2H_2_O, Na_2_MoO_4_·2H_2_O, and Na_2_B_4_O_7_·10H_2_O as one of the metal complexes. However, the hydrate with Cl^−^, OH^−^, C_6_H_5_O_7_^3−^, PO_4_^3−^, NO_3_^−^, SO_4_^2−^, and NH_4_^+^ cannot contribute to the adhesion effect. The metal that can be adhered on the MCF rubber with the hydrate is stainless steel, iron, and lead, but those that cannot be adhered are aluminum, titanium, nickel, zinc, brass, and copper. MCF rubber is composed of NR-latex or CR-latex. The anionic radical hydrate is reacted with isoprene molecules to realize cross-linking between them so that the MCF rubber with the hydrate is solidified (chemical reaction Equation (2)). By using the adhesive technique, we prepare five sensors fabricated with metal and rubber, as shown in Figure 9. We can also electrolytically polymerize an electroconductive adhesive tape. By using the present adhesive technique, we can reduce the contact resistance between the metal and rubber. In the case of the wire-type sensor, the durable tensile strength in the case of Na_2_B_4_O_7_·10H_2_O is the largest. However, in the case of the plate-type sensor, the durable tensile strength in the case of Na_2_WO_4_·2H_2_O is the largest. The former sensor is applicable to sense electrically during shear motion of the sensor, and the latter is suitable upon pressing to the sensor. The former sensor has stable sensing without perturbation. Furthermore, the former sensor is durable for tension up to about 1 kg. The latter sensor has the feasibility of agile switching effect by small pressure.

The adhesive technique proposed in the present study is applicable not only to produce MCF rubber sensor but to produce an ordinary sensor made of rubber and to realize cohesion between rubber and metal. In particular, the sensor with adhesion of the thin electric wire on the rubber, such as type III sensor, is effective to produce artificial skin made of rubber over which electric wires spread on a robot like a human skin with nerves. Furthermore, the cohesion between rubber and metal in the case of the hydrate-containing fabricated metallic or non-metallic element with more than two valency under electrolytic polymerization is novel enough to expand to vast engineering applications that involve the coating of rubber on metal surfaces to prevent corrosion.

## Figures and Tables

**Table 1:** Constituent of MCF rubber F in Figure 7.

	Type (A)	Type (B)	Type (C)	Type (D)
Ni	3	1	3	1
MF (W40)	0.75	0.75	0.75	0.75
NR-latex	3	3	3	3
CR-latex (671A)	-	-	3	3
TiO_2_	-	0.5	-	0.5

unit: g

**Table 2:** The state of adhesion by electrolytic polymerization of interfaces B and D in Figure 7; electrolytic polymerization conducted with 1 mm metal plates gap, constant electric field at 6 V, 2.7 A, and 10 min and 312 mT magnetic field generated by permanent magnets as paired opposites.

MCF Rubber F		Type (A)				Type (B)			
Electrode	A	−		+		−		+	
	C	+		−		+		−	
Surface E		CA.	CC.	CA.	CC.	CA.	CC.	CA.	CC.
Interface B		N.	N.	A.	A.	N.	N.	A.	A.
Interface D		A.	A.	A.	A.	A.	A.	A.	A.
↓ Consecutive with reversing electrodes A and C									
Electrode	A	+		−		+		−	
	C	−		+		−		+	
Interface B		N.	N.	N.	N.	N.	N.	N.	N.
Interface D		A.	A.	A.	A.	A.	A.	A.	A.

A.: Adhesion, N.: Non-adhesion, (a): 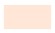

**Table 3:** Sequel to Table 2.

MCF Rubber F		Type (C)				Type (D)			
Electrode	A	−		+		−		+	
	C	+		−		+		−	
Surface E		CA.	CC.	CA.	CC.	CA.	CC.	CA.	CC.
Interface B		N.	N.	A.	A.	N.	N.	A.	A.
Interface D		A.	A.	A.	A.	A.	A.	A.	A.
↓ Consecutive with reversing electrodes A and C									
Electrode	A	+		−		+		−	
	C	−		+		−		+	
Interface B		A.	A.	A.	N.	A.	A.	N.	N.
Interface D		A.	A.	A.	A.	A.	A.	A.	A.

A.: Adhesion, N.: Non-adhesion, (a):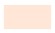; (b):
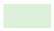

## Appendix A

Depending on the type of dopant, MCF rubber obtained by electrolytic polymerization is mainly divided into three types with different electrical properties, as shown in Table A1. The MCF rubber has induced voltage and electric current [4]. Typically, the electric resistance is small when the induced voltage is small, but the electric resistance is large when the induced voltage is large. Battery-type MCF rubber is situated as the case of having large induced electric current at piezo-typed MCF rubber, and as soft polymer battery.

**Table A1:** Three types of electrolytically polymerized MCF rubber mainly divided by kinds of dopant.

MCF Rubber Type	Induced Voltage	Induced Electric Current	Electric Resistance	Dopant
Conductive type	Minimum (1-ordered mV)	Minimum (1-ordered µA)	Minimum (0.1, 1-ordered Ω)	KI, I_2_, Tetraethylammonium tetrafluoroborate
Piezo type	Large (10, 100-ordered mV)	Small (10-ordered µA)	Large (kΩ, MΩ)	TiO_2_, ZnO, BaTiO, Aluminum nitride, Lead(II) titanium(IV) trioxide, Potassium niobate, Lithium niobate
Battery type	Large (10, 100-ordered mV)	Large (100-ordered µA)	Large (kΩ, MΩ)	KOH, Lithium hydroxide monohydrate, Trilithium Citrate Tetrahydrate

Figure A1 shows a photograph of each surface of the electrolytically polymerized MCF rubber at the anode and cathode with the application of 6 V, 2.7 A, and 188 mT and with a 1 mm space between the electrodes by conventional graduated about ×300 light microscope. The MCF rubber consisted of 12 g Ni powder, 3 g water-based MF with 50 wt.% Fe_3_O_4_ (M-300, Sigma Hi-Chemical Co. Ltd., Tsutsujigasaki, Japan) and 12 g NR-latex [1]. Its surface on the cathode side was concave and convex, and that on the anode side was smooth. The rubber is vulcanized to grow incrementally out from the anode surface.

The MCF rubber liquid without a hydrate is electrolytically polymerized between different kinds of electrode metals, as shown by Figure A2. The MCF rubber liquid was composed of 3 g carbonyl Ni powder, 0.75 g water-based MF (W-40), and 3 g NR-latex. The liquid was poured between each metal plates with a 1 mm gap, and a constant electric field was applied at 6 V; an electric current of 2.7 A was passed between the plates for 15 min and application of a 312 mT magnetic field by permanent magnets as paired opposites across the liquid. Figure A2 shows each anode and cathode separated into parts from facing state each other after the electrolytic polymerization. The electrolytically polymerized MCF rubber in the case of aluminum cannot be easily detached from the electrodes than that in any other kinds of electrode metal. Rubber can be the most smoothly detached from the stainless steel electrodes than from any other kinds of electrode metals, so stainless steel is most suitable for obtaining electrolytically polymerized MCF rubber for engineering applications.

In carboxyl group of oleic acid coated around Fe_3_O_4_, electron transfers to become ionized by the mesomeric effect [24] as shown by Equation (A1).

(A1)
